# A Case Report of Long-Term Survival Achieved by Nivolumab and Hyperthermia With Multiple Local Therapies for the Peritoneal Dissemination of Gastric Cancer

**DOI:** 10.7759/cureus.69147

**Published:** 2024-09-11

**Authors:** Takayuki Ohguri, Subaru Tani, Yo Kawarada, Masahiro Kitahara

**Affiliations:** 1 Therapeutic Radiology, Hospital of the University of Occupational and Environmental Health, Kitakyushu, JPN; 2 Gastroenterological Surgery, National Hospital Organization Kanmon Medical Center, Shimonoseki, JPN

**Keywords:** gastric cancer, hyperthermia, nivolumab, oligometastases, peritoneal dissemination

## Abstract

A 60-year-old male with recurrent metastatic gastric cancer achieved long-term survival with nivolumab, hyperthermia, and local multisite therapy. The patient had a history of multiple relapses despite receiving standard treatment. After the failure of multiple lines of chemotherapy, nivolumab and hyperthermia were initiated. During this combination therapy, local treatments including surgery and radiation therapy were administered to treat the progressive disease. Remarkably, the patient achieved more than five years of overall survival time after starting nivolumab and external repeated hyperthermia with local therapies and has shown no measurable disease on imaging for the past 24 months. This case suggests that a combination of nivolumab, hyperthermia, and local therapies may offer a potential therapeutic strategy for patients with advanced gastric cancer.

## Introduction

Nivolumab is effective in patients with advanced or recurrent gastric cancer who progress after standard chemotherapy [1]. However, the limited overall survival benefit of approximately five months necessitates further exploration of therapeutic strategies to improve patient outcomes. Combining hyperthermia, which reportedly has a cancer-specific immune-activating effect mediated by heat shock proteins (HSPs), with radiation therapy, which is known to exhibit an immune-stimulatory effect, has shown promise as a multimodal treatment approach [2-4]. Takeda et al. reported promising clinical results for the combined treatment of hyperthermia with immune checkpoint inhibitors [5].

Here, we present the case of a patient with peritoneal dissemination of gastric cancer who achieved long-term survival exceeding five years following treatment with a combination of nivolumab, hyperthermia, and multisite local therapies, including radiotherapy and surgery.

## Case presentation

A 60-year-old male had undergone gastrectomy for gastric cancer eight years previously. The pathological stage was pT4aN2M0 and the tumor was a moderately differentiated tubular adenocarcinoma. Adjuvant chemotherapy with cisplatin (CDDP) and S-1 was initiated but was discontinued after two weeks due to abdominal pain caused by S-1. Peritoneal dissemination occurred six years and six months prior, and CDDP + S-1 was started again but was discontinued due to a recurrence of abdominal pain. Radiation therapy (41.4 Gy/23 fractions) was administered for perigastric lymph node metastasis and disseminated lesions around the gallbladder five years and eight months previously. Second-line chemotherapy with CPT-11 was started five years and two months prior, but was discontinued after two months due to diarrhea.

**Figure 1:**
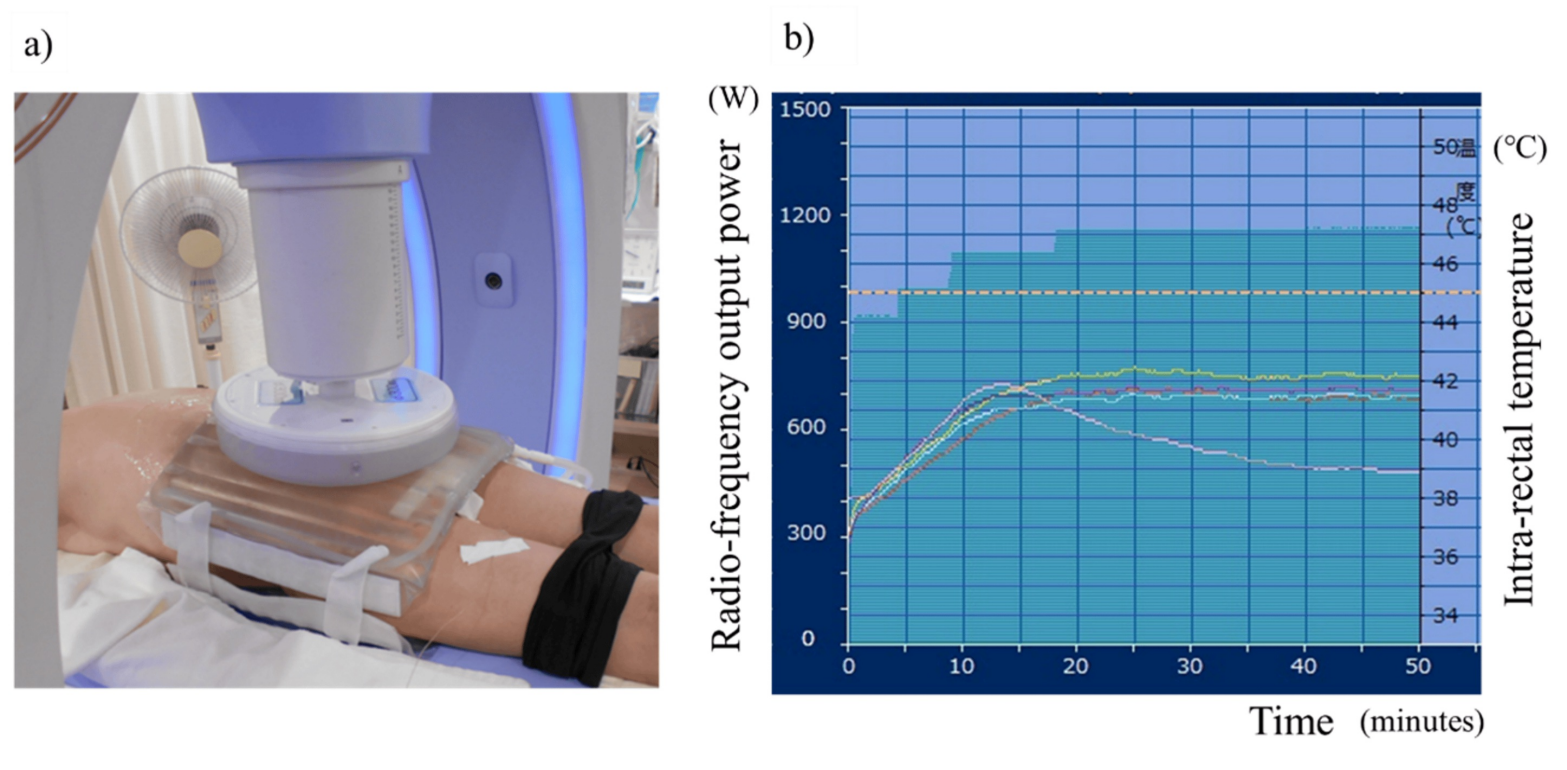
Hyperthermia through external electromagnetic heating. (a) Settings for deep regional hyperthermia for the pelvic region using the 8 MHz radiofrequency capacitive heating device. The regular bolus, which attaches to electrodes of 30 cm in diameter, and the overlay bolus, in which internal cooled ﬂuid circulates and cools the skin surface broadly. (b) Changes in intra-rectal temperature and RF power during a single session of hyperthermia in the pelvic region. The red line indicates the temperature measured in the rectum at 7 cm from the anus, the yellow line at 5.5 cm, the light blue line at 4 cm, and the purple line at 2.5 cm. The skin-colored line is the surface temperature of the skin at the buttock cleft. The temperature in the rectum reaches 41-42 °C within 20 minutes after the start of heating and is maintained until 50 minutes after heating. The thermal dose (CEM43T90) for this heating session is 3.5 minutes. The green-filled range indicates changes in RF output. RF: radiofrequency

Third-line nivolumab (240 mg every two weeks) was initiated five years ago. Hyperthermia was induced once per week during nivolumab administration (Thermotron RF-8TM; Yamamoto Vinita, Osaka, Japan). Nivolumab was administered approximately three hours after hyperthermia treatment. This timing was chosen based on previous studies demonstrating that hyperthermia can induce upregulation of PD-L1 and HLA class I molecules [5,6]. The physical features of this instrument, including its thermal distribution in a phantom model and in the human body, have been described previously [7,8]. The median heating time was 50 minutes according to patient tolerance. Figure 1 details the hyperthermia treatment.

**Figure 2:**
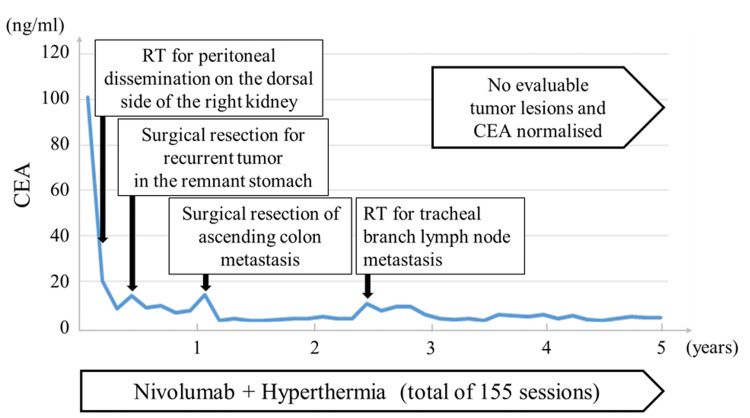
Time course of carcinoembryonic antigen (CEA) changes and timing of additional local therapies after the start of combination therapy with nivolumab and hyperthermia RT: radiation therapy

Radiofrequency (RF) output power was increased to the maximum power tolerated by the patient and maintained with a target of 42˚C, based on correlative data between RF output power and deep regional temperature [9]. Hyperthermia was induced immediately prior to nivolumab infusion. Brieﬂy, for hyperthermia treatment, both the upper and lower electrodes were 30 cm in diameter. The pelvic and abdominal regions were alternately heated weekly. The combined treatment resulted in a marked decrease in carcinoembryonic antigen (CEA) levels (Figure 2).

**Figure 3:**
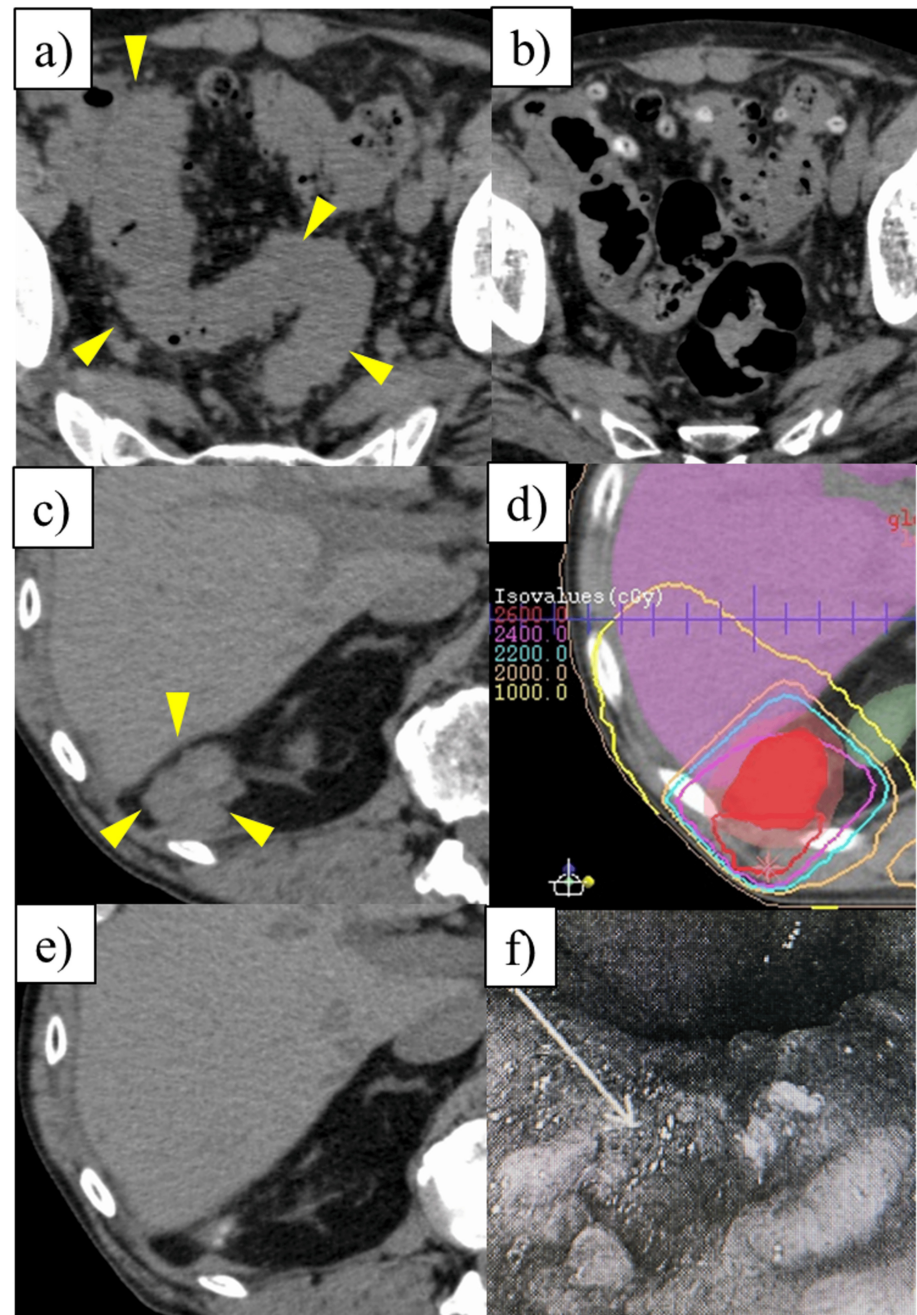
Course of the disseminated metastatic and recurrent disease. (a) CT image of pelvic dissemination before initiation of combined nivolumab and hyperthermia treatment. (b) CT image four months after the start of combined treatment with nivolumab and hyperthermia showing disappearance of pelvic dissemination. (c) CT image of the peritoneal dissemination on the dorsal side of the right kidney one month after the start of nivolumab and hyperthermia. (d) Dose distribution of radiotherapy for the peritoneal dissemination on the dorsal side of the right kidney (24 Gy/6 fractions), aiming to enhance the therapeutic efficacy of nivolumab. (e) CT images four months after completion of radiotherapy show metastatic disappearance. (f) Endoscopic photograph of the recurrent tumor in the remnant stomach.

During combination therapy, additional radiation therapy (24 Gy/6 fractions) was administered for peritoneal dissemination to the dorsal side of the right kidney to enhance the therapeutic efficacy of nivolumab (Figure 3).

Consequently, the pelvic peritoneal disseminations visible on CT disappeared four months after the initiation of nivolumab and hyperthermia. In subsequent years, nivolumab and hyperthermia were continued, and as CEA levels increased, newly developed metastatic lesions were treated with either radiotherapy or surgical resection. Total gastrectomy for the recurrent tumor in the remnant stomach was performed four years and six months ago, and resection of the ascending colon metastasis was performed four years ago; the pathology of the excised specimens showed a moderately differentiated adenocarcinoma consistent with the primary tumor.

Additional radiation therapy (30 Gy/10 fractions) was administered for metastatic lesions in the tracheal bifurcation lymph nodes one year and nine months previously. The evolution of CEA levels after nivolumab and hyperthermia administration and the timing of the addition of the above local therapies are also shown in Figure 2. Nivolumab and hyperthermia have been continued (total of 155 sessions) to date. The patient has maintained no measurable disease on imaging for the past 24 months, and the tumor markers have normalized. The patient has survived for five years since the initiation of nivolumab therapy.

## Discussion

In one randomized clinical trial, nivolumab significantly prolonged overall survival for patients with unresectable advanced or recurrent gastric cancer or adenocarcinoma of the esophagogastric junction that is refractory or intolerant to standard chemotherapy, and is now widely used in daily clinical practice [1]. In the 330 patients who recieved the nivolumab in the randomized clinical trial, the median overall survival was only 5.26 months. Therefore, the exceptionally favorable outcome in our case is difficult to explain solely based on nivolumab administration.

There have been clinical reports on hyperthermic intraperitoneal chemotherapy (HIPEC) as an aggressive treatment for gastric cancer with peritoneal metastasis. In the Phase III GASTRIPEC-I- study, HIPEC for gastric cancer with peritoneal metastases combined with cytoreductive surgery was used; HIPEC comprised mitomycin C 15 mg/m^2^ and cisplatin 75 mg/m^2^ in 5 L of saline perfused for 60 minutes at 42°C [10]. The median overall survival was 14.9 months, and the addition of HIPEC resulted in significantly better progression-free and distant metastasis-free survival rates. In 2019, Yarema et al. reported a retrospective cooperative study among six central-eastern European HIPEC centers [11]. Cytoreductive surgery and HIPEC resulted in a median overall survival of 12.6 months in 70 patients with limited overt peritoneal metastases. However, none of the 70 patients in the HIPEC study survived for more than five years. While the combination of HIPEC and cytoreductive surgery shows promise, long-term survival exceeding 6.5 years, as observed in our case, is typically not achievable. Therefore, other contributing factors are likely to have been involved in the outcomes of our case.

One of the presumed reasons for the favorable results in our case was the activation of the immune system induced by hyperthermia. Hyperthermia with external heating is non-invasive, synchronized with nivolumab administration, and has the advantage of being repeatable. As noted above, the efficacy of nivolumab in patients with advanced gastric cancer is limited, with an overall survival of only 5.3 months [1]. The elucidation of the resistance mechanisms of immune checkpoint inhibitors and the development of sensitizing agents are urgently needed in clinical practice. A characteristic feature of cases resistant to immune checkpoint inhibitors is the lack of immune cell infiltration into the tumor and tumors that do not express immune checkpoint proteins (so-called cold tumors), which lack an inflammatory response. Therapeutic tools that enhance immune cell infiltration and immune checkpoint protein expression in cold tumors are considered potential sensitizers to immune checkpoint inhibitors by converting cold tumors into hot tumors. A number of preclinical reports have shown that hyperthermia not only induces direct antitumor effects on cancer cells but is also known to activate tumor immunity [2,3,12-14]. Heat-induced immune activation can be summarized as follows: Heat exposure triggers a multifaceted immune response. It induces the expression of HSPs, such as HSP60, HSP70, and HSP90, which function as chaperones and danger signals and promote immune cell activation. Additionally, heat enhances antigen presentation by upregulating major histocompatibility complex (MHC) molecules and co-stimulatory signals in antigen-presenting cells (APCs), leading to efficient T-cell activation. Furthermore, heat activates the nuclear factor kappa-light-chain-enhancer of activated B cells (NF-κB) pathway, driving the production of inflammatory cytokines and immune modulators, and promoting immune cell recruitment and activation. Finally, heat directly stimulates NK cells, thereby enhancing their cytotoxicity and cytokine production.

Kubota et al. reported that, in clinical cases and animal models, immunohistochemical staining showed that the expression of PD-L1 and MHC class I, as well as the invasion of CD8 + cells, increased after hyperthermia [5]. Takeda et al. presented two case studies demonstrating remarkable responses in patients with non-small cell lung cancer and pancreatic cancer treated with a combination of nivolumab, hyperthermia, and dendritic cell therapy [15]. In addition, a clinical case report has shown that low-dose chemotherapy combined with regional hyperthermia in patients with rhabdomyosarcoma with multiple metastases resulted in complete remission not only in the heated area but also in distant metastases and that this effect was related to the activation of NK and T cells [16].

The relationship between radiotherapy and cellular immune responses has garnered significant attention in recent years. Radiation therapy unexpectedly bolsters antitumor immunity through various mechanisms beyond its direct cytotoxic effects on tumor cells. It induces tumor cell death, leading to the release of antigens that stimulate CD8+ T cells upon presentation by APCs. Recent reports have demonstrated that ionizing radiation induces immunogenic tumor cell death in distant non-irradiated lesions through tumor-specific cytotoxic T lymphocyte responses [17,18]. Radiation can also modulate immune checkpoints, thereby increasing the vulnerability of tumor cells to immunotherapy. Furthermore, radiation directly activates dendritic cells, potentiating T-cell responses. These interrelated mechanisms underlie the synergistic effects observed when radiotherapy is combined with cancer immunotherapy.

In our case, the combination of nivolumab and repeatable external hyperthermic heating was conducted over a long period of 155 sessions. In addition, the combination with radiotherapy may have stimulated the tumor-specific immune response described above and inhibited the development of new metastatic lesions in the long term. We speculate that hyperthermia- and radiotherapy-induced immunogenic effects play a role in the suppression of metastases. Response to treatment and host immune status may be key factors in the clinical relevance of micrometastases. In the current case, repeated hyperthermia and radiotherapy for the metastases may have suppressed the clinical expression of micrometastases.

## Conclusions

This was a very rare case of gastric cancer with peritoneal dissemination that achieved more than five years of overall survival time after the start of nivolumab plus external repeated hyperthermic treatments and local therapies. The current case is significant from a clinical perspective, highlighting the importance of nivolumab plus hyperthermia with local therapies to treat the peritoneal dissemination of gastric cancer.
